# The impacts of surgical mask in young healthy subjects on cardiopulmonary function and muscle performance: a randomized crossover trial

**DOI:** 10.1186/s13690-022-00893-4

**Published:** 2022-05-17

**Authors:** Haining Ou, Yuxin Zheng, Mei Li, Junjie Liang, Hongxin Chen, Shijuan Lang, Qinyi Li, Delong Chen, Youwei Lin, Qiuxia Chen, Yue Sun, Meifeng Zheng, Tingting You, Qiang Lin

**Affiliations:** 1 Department of Rehabilitation Medicine, The Fifth Affiliated Hospital of Guangzhou Medical University, No. 621, Gangwan Road, Huangpu District, Guangzhou, 510700 China; 2 The Rehabilitation Medicine Lab, The Fifth Clinical College of Guangzhou Medical University, Guangzhou, 510700 China; 3 Guangzhou Key Laboratory of Enhanced Recovery after Abdominal Surgery, The Fifth Affiliated Hospital of Guangzhou Medical University, Guangzhou, 510700 China; 4 Department of Rehabilitation Therapy, Guangzhou Medical University, Guangzhou, 511436 China; 5 Department of Geriatrics, The Fifth Affiliated Hospital of Guangzhou Medical University, Guangzhou, 510700 China

**Keywords:** CPET, sEMG, Surgical masks, Respiratory infectious disease, Cardiopulmonary function

## Abstract

**Objective:**

To explore the impacts of surgical mask in normal subjects on cardiopulmonary function and muscle performance under different motor load and gender differences.

**Design:**

Randomized crossover trial.

**Setting:**

The Fifth Affiliated Hospital of Guangzhou Medical University, June 16th to December 30th, 2020.

**Participants:**

Thirty-one college students (age: male 21.27 ± 1.22 years; female 21.31 ± 0.79 years) were recruited and randomly allocated in two groups.

**Interventions:**

Group 1 first received CPET in the mask-on condition followed by 48 h of washout, and then received CPET in the mask-off condition. Group 2 first received CPET in the mask-off condition followed by 48 h of washout, then received CPET in the mask-on condition. The sEMG data were simultaneously collected.

**Main outcome measures:**

The primary outcome was maximum oxygen uptake (VO_2_ max) from CPET, which was performed on a cycle ergometer—this is the most important parameter associated with an individual’s physical conditioning. The secondary parameters included parameters reflecting exercise tolerance and heart function (oxygen uptake, anaerobic valve, maximum oxygen pulse, heart rate reserve), parameters reflecting ventilation function (respiration reserve, ventilation volume, tidal volume, breathing frequency), parameters reflecting gas exchange (end-tidal oxygen and carbon dioxide partial pressure, oxygen equivalent, carbon dioxide equivalent, and the relationship between dead space and tidal volume) and parameters reflecting skeletal muscle function [oxygen uptake, anaerobic valve, work efficiency, and EMG parameters including root mean square (RMS)].

**Results:**

Comparing the mask-on and mask-off condition, wearing surgical mask had some negative effects on VO_2_/kg (peak) and ventilation (peak) in both male and female health subjects [VO_2_/kg (peak): 28.65 ± 3.53 vs 33.22 ± 4.31 (*P* = 0.001) and 22.54 ± 3.87 vs 26.61 ± 4.03 (*P* < 0.001) ml/min/kg in male and female respectively; ventilation (peak): 71.59 ± 16.83 vs 82.02 ± 17.01 (*P* = 0.015) and 42.46 ± 10.09 vs 53.95 ± 10.33 (*P* < 0.001) liter in male and female respectively], although, based on self-rated scales, there was no difference in subjective feelings when comparing the mask-off and mask-on condition. Wearing surgical masks showed greater lower limb muscle activity just in male subjects [mean RMS of vastus medialis (load): 65.36 ± 15.15 vs 76.46 ± 19.04 μV, *P* = 0.031]. Moreover, wearing surgical masks produced a greater decrease in △tidal volume (VTpeak) during intensive exercises phase in male subjects than in female [male − 0.80 ± 0.15 vs female − 0.62 ± 0.11 l *P* = 0.001].

**Conclusions:**

Wearing medical/surgical mask showed a negative impact on the ventilation function in young healthy subjects during CPET, especially in high-intensity phase. Moreover, some negative effects were found both in ventilation and lower limb muscle actives in male young subjects during mask-on condition. Future studies should focus on the subjects with cardiopulmonary diseases to explore the effect of wearing mask.

**Trial registration:**

Chinese Clinical Trial Registry (ChiCTR2000033449).

## Background

Wearing mask is an effective way to prevent respiratory diseases in daily life, especially for respiratory infectious diseases during the pandemic [1]. The efficacy of public health measures to control the transmission of severe acute respiratory diseases is not certain, thus, wearing a mask outside and inside in public might be normalized for a long time. Compared with respirators (which just recommended to use by medical, wearing surgical mask is more recommended for health people in the daily life to reduce transmissions from the wearer to the patient or contact with large infectious droplets due to its easy availability, comfort, and compliance with community requirements [2]. So, all the medical masks mentioned in this study referred to the surgical masks.

Wearing mask could reduce the risk of contracting contagious respiratory infections [3, 4], but its direct physical effect hinders gas exchange at the same time, which might affect human motor performance under mask-on conditions, and even safety under high-intensity exercise.

The relevant research is extremely limited. For example, in Sven Fikenzer’s and Davies’s studies, ventilation and cardiopulmonary exercise capacity were reduced by surgical masks in 9and 12 healthy males, respectively [5, 6]. The studies published so far have each recruited only a small number of male subjects with a relatively large age span; however, age and gender differences are important factors affecting exercise and cardiopulmonary function [7, 8]. Meanwhile, none of the published studies have addressed the effect of wearing mask on the direct motor effector (muscle performance).

In this study, we used the cardiopulmonary exercise testing (CPET) and lower limber simultaneous surface electromyography (sEMG) to explore the impacts of surgical mask in normal subjects on cardiopulmonary function and muscle performance under different motor load and gender differences.

## Methods

### Trial design

The study was designed as a randomized cross-over study to explore the impacts of surgical mask in normal subjects on cardiopulmonary function and muscle performance under different motor load and gender differences, which has been registered at the China Clinical Trial Registration Center (No. ChiCTR2000033449).

### Participants

Subjects were recruited from college students at Guangzhou Medical University. The inclusion criteria were: subjects between 18 and 26 years old; who could pass the Physical Activity Readiness Questionnaire (PAR-Q) test [9]; were physically healthy; without professional sports training experience; and were able to understand the experiment and voluntarily cooperate with the whole process of the test. The exclusion criteria were: cardiovascular diseases and respiratory diseases; subjects with lower limb motor dysfunction caused by other diseases; subjects who could not cooperate with the experiment; smokers and any other contraindications to CPET [10]. A total of 35 subjects were recruited through electronic posters, and travel expenses were reimbursed. Written informed consent was obtained from all subjects.

### Interventions

**Fig. 1:**
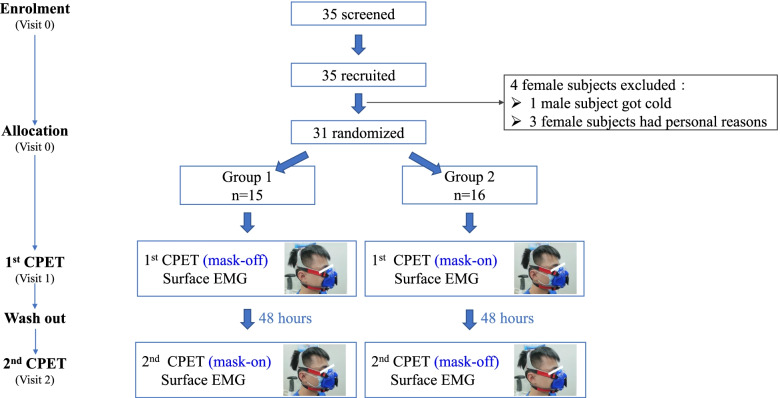
Study design and recruitment

The participants were randomly allocated into two groups using a digit table: Group 1 first received CPET in the mask-on condition followed by 48 h of washout, and then received CPET in the mask-off condition. Group 2 first received CPET in the mask-off condition followed by 48 h of washout, then received CPET in the mask-on condition. The sEMG data were simultaneously collected. The design and recruitment of the study are shown in Figs. 1 and 3A.

#### Surgical/medical masks

The typical and widely used disposable surgical mask with ear loops (Haozheng Weicai, Haozheng Sanitary Materials Factory, Guangzhou, China) was used in the mask-on condition during the CPET test.

#### Subject preparation

The laboratory temperature was set at 25 °C, and the instrument was calibrated before each test. Subject preparation including as follows: eating only a light meal at least two hours prior to the procedure, avoiding carbonated or caffeinated drinks, as well as alcohol, avoiding exercise at the day of the test, and dressing in comfortable clothes.

#### Spirometry testing

**Fig. 2:**
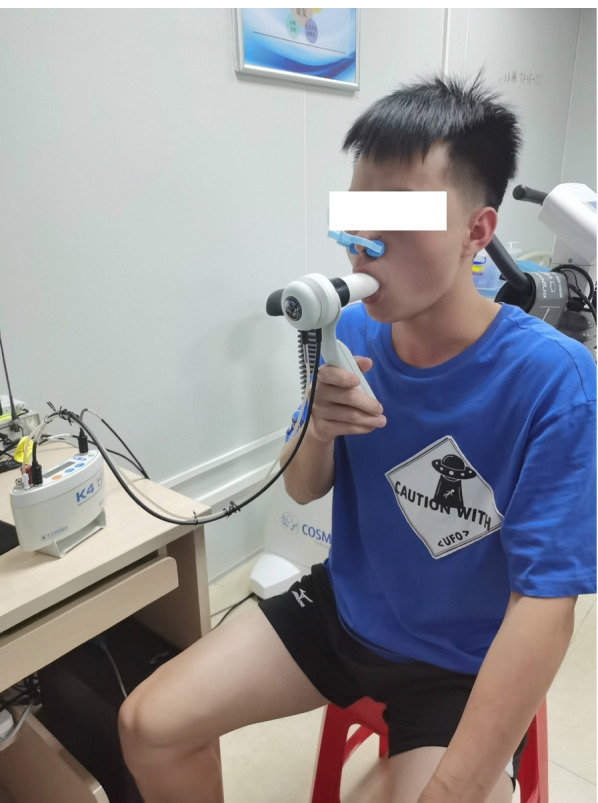
Spirometry testing for evaluating baseline lung function

The participant was instructed to take a deep breath in, hold the breath for a few seconds, and then exhale as hard as he/she could into the breathing mask, which was used to test the baseline lung function in the mask-off condition (Fig. 2). The participant was asked to repeat this test at least three times to make sure the results were consistent. Forced vital capacity (FVC) and forced expiratory volume in 1 second (FEV1) were collected as the two main parameters to measure airflow into and out of the lungs. The FEV1/FVC ratio was calculated and used as an exclusion criterion (if less than 70%, which indicates a relatively low lung function) [11].

#### Cardiopulmonary exercise tests (CPETs)

**Fig. 3:**
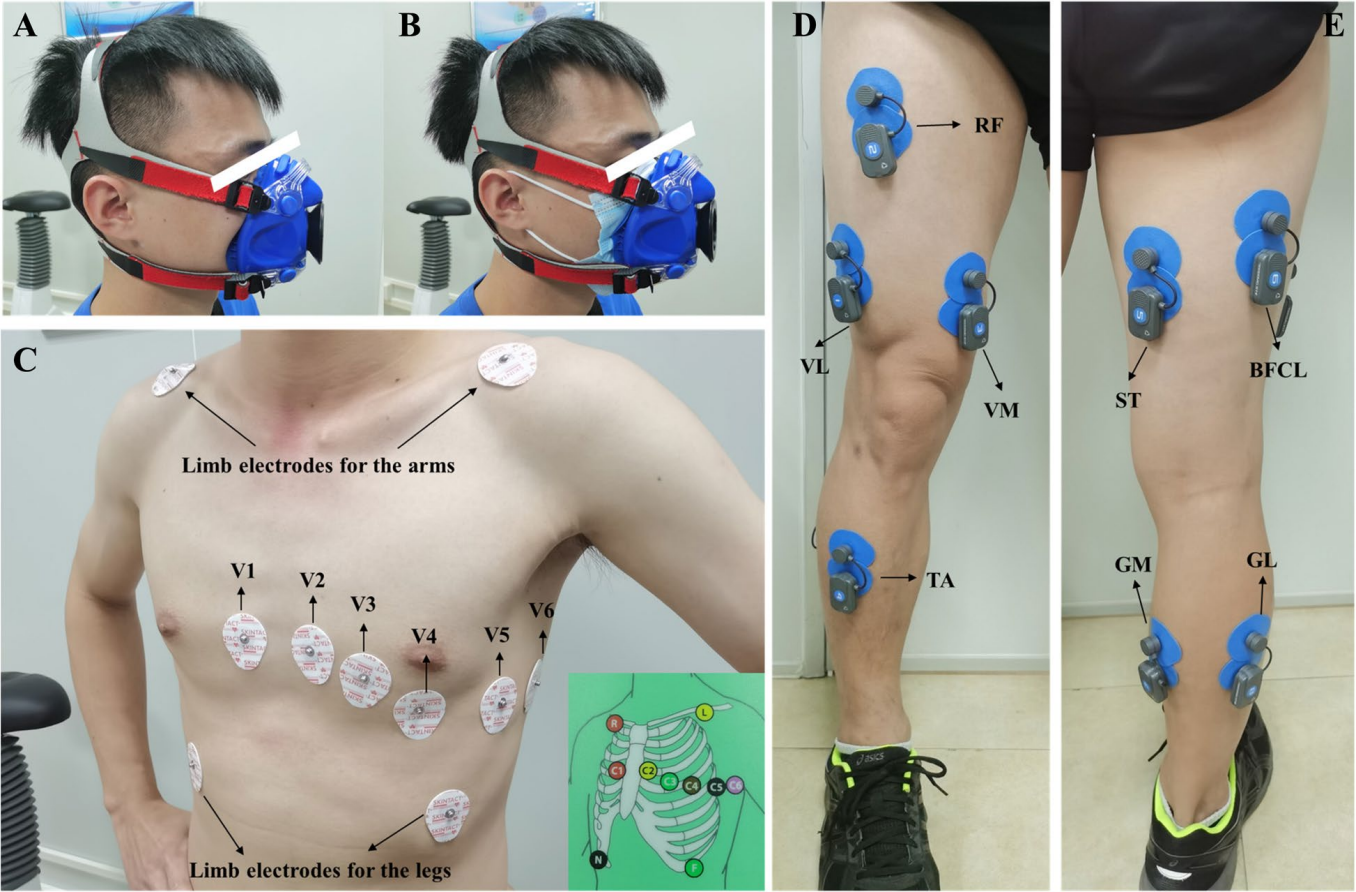
Subject preparation including mask-on condition (**A**), mask-off condition (**B**), V1-V6 ECG settings (**C**) and EMG settings on the front view (**D**) and behind view (**E**)

**Fig. 4:**
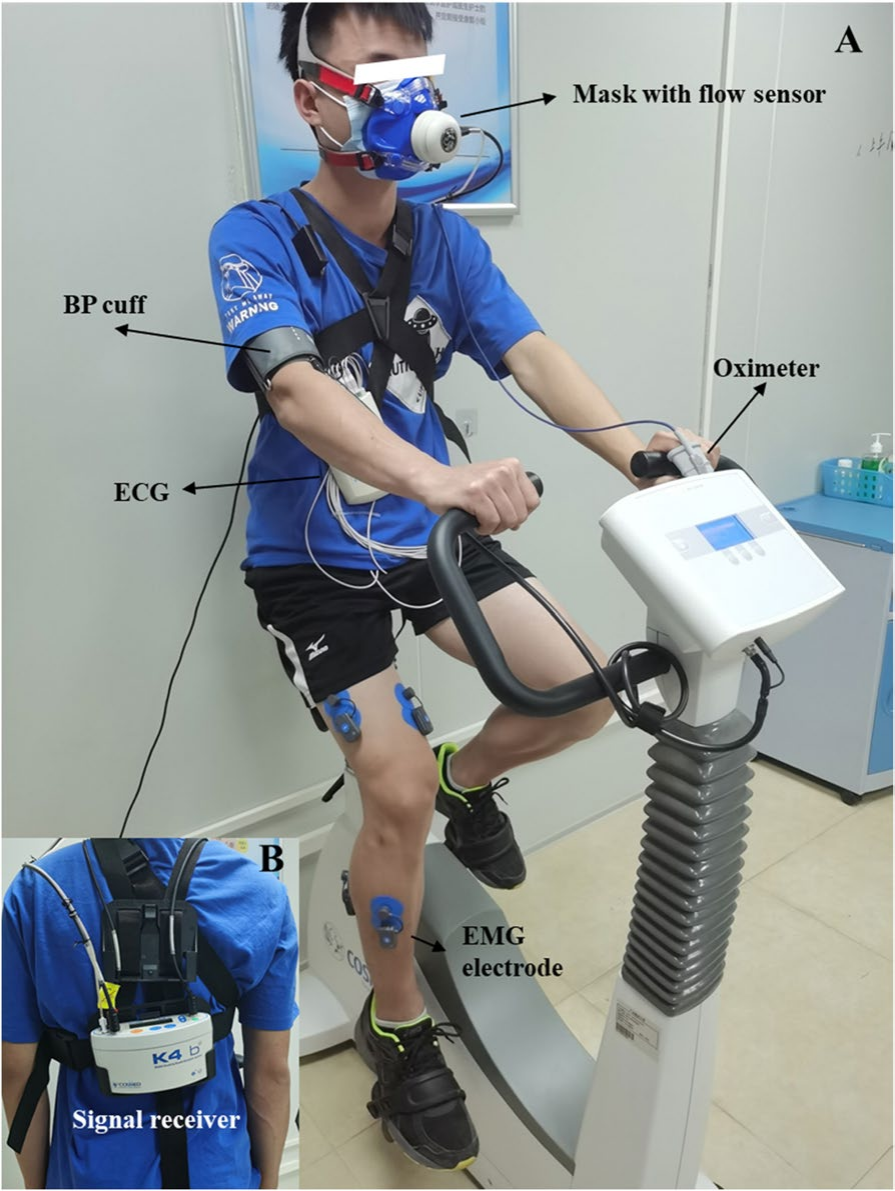
CPET setting. Front view (**A**) and back view (**B**)

The mode of cycle ergometer exercise and progressive incremental exercise (ramp protocol) were used in the CPET protocol, which consists of 2 min of the resting stage, followed by 2 min of the unloaded pedaling stage (Unloaded stage: 0 watt/min), followed by 8–12 min of incremental/ramp exercise stage [Loaded stage: increased by ramp (watt/min), and the ramp was based on the formula as follow] until the subjects reach volitional exhaustion or the test is terminated by the medical monitor (see criteria for terminating the exercise test). This CPET study protocol was referred with our previous study [12]. Then, the participant ended with 3 min of recovery stage (0 watt/min) and 3 min of observation stage (static sitting). 12-lead electrocardiography (ECG) (Fig. 3B), pulse oximetry, and blood pressure were tracked to detect any abnormalities during the process (Fig. 4).

The formulas below were used for calculating the CPET performance for each participant [13]:

$$\mathrm{Ramp}=\left[{\mathrm{VO}}_{2\mathrm{peak}}-{\mathrm{VO}}_{2\mathrm{unloaded}}\right]/100$$

$$\mathrm{Ramp}\ \left(\mathrm{male}\right)=\left[\left(\mathrm{height}-\mathrm{age}\right)\ast 20-\left(150+6\ast \mathrm{weight}\right)\right]/100$$

$$\mathrm{Ramp}\ \left(\mathrm{female}\right)=\left[\left(\mathrm{height}-\mathrm{age}\right)\ast 14-\left(150+6\ast \mathrm{weight}\right)\right]/100$$

#### Criteria for terminating the exercise test

Based on the American Thoracic Society/American College of Chest Physicians Guidelines, CPET is a relatively safe procedure, especially in healthy individuals. This study followed the criteria for exercise termination before symptom limitation, as follows: chest pain suggestive of ischemia, ischemic ECG changes, complex ectopy, second- or third-degree heart block, fall in systolic pressure 20 mmHg from the highest value during the test, hypertension (250 mmHg systolic; 120 mmHg diastolic), severe desaturation: SpO_2_ 80% when accompanied by symptoms and signs of severe hypoxemia, sudden pallor, loss of coordination, mental confusion, dizziness or faintness, and signs of respiratory failure [10].

#### Post-CPET assessments

The Borg category ratio scale (Borg CR-10 scale) [14] was performed after every CPET (mask-on CPET and mask-off CPET), which was used for reflecting the intensity load of performing the cycle-ergometer exercise.

#### Simultaneous surface electromyography (sEMG)

sEMG data were simultaneously collected using the BTS FREEEMG 1000 (BTS Bioengineering Spa, Italy) device with surface electrodes during the CPET. The electrodes were attached to the tibialis anterior (TA), lateral gastrocnemius (LG), medial gastrocnemius (MG), rectus femoris (RF), vastus medialis (VM), vastus lateralis (VL), semitendinosus (ST), and biceps femoris caput longus (BFCL) in the dominant lower limb (Fig. 3C and D).

### Outcomes

The primary outcome was maximum oxygen uptake (VO_2_ max) from CPET, which was performed on a cycle ergometer—this is the most important parameter associated with an individual’s physical conditioning. The secondary parameters included parameters reflecting exercise tolerance and heart function (oxygen uptake, anaerobic valve, maximum oxygen pulse, heart rate reserve), parameters reflecting ventilation function (respiration reserve, ventilation volume, tidal volume, breathing frequency), parameters reflecting gas exchange (end-tidal oxygen and carbon dioxide partial pressure, oxygen equivalent, carbon dioxide equivalent, and the relationship between dead space and tidal volume) and parameters reflecting skeletal muscle function [oxygen uptake, anaerobic valve, work efficiency, and EMG parameters including root mean square (RMS)].

### Sample size estimation

The number of subjects required in this cross-control trial study was calculated to ensure adequate statistical effectiveness. The value of VO_2_ peak was used as the main outcomes. The mean and standard deviation of five subjects in mask-on and mask-off were 1326.2 ± 123.2 and 1091.6 ± 187.6, respectively. The sample size was calculated using G-Power 3.1.9. We set effect size of 0.39 and a power of 0.95 for determining the required minimum sample size of nine.

### Data collection and management

CPET data during the unloaded pedaling stage and incremental/ramp exercise stage were exported from the CPET equipment into an Excel file for statistical analysis. sEMG data were analyzed using the Smart EMG software (BTS system-provide). A 300 Hz low-pass filter and a 10 Hz high-pass filter were used by Hamming filter, and then full-wave rectification was performed, taking 100 ms as the time window for root means square (RMS) amplitude value to intercept the unload and load signals, respectively. The mean of unloading and loading were calculated for final statistical analyses.

### Statistical analysis

All the data were analyzed using SPSS software (version 25, IBM Crop., Armonk, NY, USA) and figures made using GraphPad Prism 8 (GraphPad Software Inc., California, USA), as described in previous studies [15, 16]. The data were expressed as means and standard deviations, and the significance level was defined as *P* < 0.05. The paired t-test was used for comparing differences of CPET and sEMG parameters between the mask-on condition and mask-off conditions. An independent sample t-test was used for comparing the differences between male and female subjects.

## Results

The subjects in this study were recruited from college students at Guangzhou Medical University from June 16th to December 30th, 2020. Thirty-five college students were recruited in this study, and four subjects were excluded (one male subject getting cold after the first CPET and three female subjects with personal reasons after the first CPET). Thirty-one subjects completed the whole study (15 males and 16 females). None of them reported any important harms or unintended effects. The recruitment information of the study are shown in Fig. 1. There were no significant differences showed in age and body mass index (BMI) between the male group and the female group. The average age was 21.27 ± 1.22 years old in the male group and 21.31 ± 0.79 years old in the female group (*P* = 0.902). The average BMI was 21.33 ± 2.74 kg/m^2^ in the male group and 20.02 ± 2.70 kg/m^2^ in the female group (*P* = 0.190). The male group showed significantly higher forced vital capacity (FVC) and higher forced expiratory volume in 1 second (FEV1) compared with the female group. The FVC was 4.43 ± 0.38 l in the male group and 3.10 ± 0.47 l in the female group (*P* < 0.001). The FEV1 was 3.85 ± 0.32 l in the male group and 3.10 ± 0.47 l in the female group (*P* < 0.001). However, the ratio of FEV1/FVC showed no significant difference between the male group and the female group (Table 1).

**Table 1:** Baseline characteristics and spirometry results in male group and female group, respectively

Parameters	Unit	Male group	Female group	***P*** value
		(***n*** = 15)	(***n*** = 16)	
**Age**	years	21.27 ± 1.22	21.31 ± 0.79	0.902
**Height**	cm	175.00 ± 6.65	159.50 ± 4.05	**< 0.001**
**Weight**	kg	65.57 ± 10.87	50.96 ± 7.50	**< 0.001**
**BMI**	kg/m^2^	21.33 ± 2.74	20.02 ± 2.70	0.190
**Spirometry**				
FVC	liter	4.43 ± 0.38	3.10 ± 0.47	**< 0.001**
FEV1	liter	3.85 ± 0.32	2.70 ± 0.31	**< 0.001**
FEV1/FVC	%	0.87 ± 0.06	0.88 ± 0.06	0.852
**General performances (mask-off condition)**				
**Load peak**	watt	193.80 ± 37.06	109.56 ± 18.23	**< 0.001**
**Exercise duration**	sec	502.40 ± 85.48	515.63 ± 78.45	0.665

*P* value stands for the comparison between male group and female group. Significant results are indicated in bold

*Abbreviations*: *BMI* Body mass index, *FVC* Forced vital capacity, *FEV1* Forced expiratory volume in 1 second, *cm* centimeter, *kg* kilogram, *m* meter, *sec* seconds

### Primary outcome

Table 2 showed the CPET parameters of within-group comparisons between mask-on condition and mask-off condition in male group and female group, respectively. For exercise tolerance and cardiac functions, comparing with mask-off condition, the male and female groups showed significantly decreased VO_2_/kg (ml/min/kg) (in unload stage, peak point, and anaerobic threshold/AT point), decreased VO_2AT_/VO_2_max (%), decreased O_2_/HR (ml/kg/beat) (in unload stage and peak point), decreased △VO_2_/△WR [ml/(min*watt)], and decreased MET (in unloading stage and peak point) in mask-on condition. At the same time, the male and female groups showed no significant differences in HR (in unload stage and at peak point) and HRR (heart rate reserve) when comparing mask-on and mask-off conditions. Also, compared with the mask-off condition, the male group showed no significant difference in SpO_2_ (%) in unload stage with mask-on condition, but decreased SpO_2_ (%) at peak point. Whereas the female group showed no significant differences in SpO_2_ (%) – neither in unload stage nor at peak point – between mask-on and mask-off conditions. For ventilatory function and gas exchange parameters, compared with mask-off condition, the male group and the female group showed significantly increased BR% (in unload stage and peak point) with mask-on condition. Moreover, the male group and the female group showed significantly decreased V_E_ (in unload stage and peak point) with mask-on condition. Only the male group showed significantly increased V_E_/VCO_2_ (slope) with mask-on condition. For CPET performance, there were no significant differences in Load _max_ (watt), exercise duration, or Borg’s scales between mask-off and mask-on conditions. There was no significant difference in exercise time for male between mask-on and mask-off, while there was no significant difference in exercise time for female between mask-on and mask-off either.

**Table 2:** The CPET parameters of within-group comparisons between mask-on condition and mask-off condition in male group and female group, respectively

Parameters	Unit	Male group			Female group		
		Mask-on	Mask-off	***P***_***1***_	Mask-on	Mask-off	***P***_***2***_
**Exercise tolerance and cardiac function**							
VO_2_/kg (unload)	ml/min/kg	7.60 ± 1.40	9.27 ± 1.71	**< 0.001**	6.75 ± 0.76	9.13 ± 1.55	**< 0.001**
VO_2_/kg (peak)	ml/min/kg	28.65 ± 3.53	33.22 ± 4.31	**0.001**	22.54 ± 3.87	26.61 ± 4.03	**< 0.001**
VO_2_/kg (AT)	ml/min/kg	18.93 ± 2.98	21.24 ± 3.60	**0.001**	15.45 ± 2.18	19.39 ± 3.07	**< 0.001**
VO_2AT_/VO_2max_	%	40.13 ± 5.55	45.13 ± 6.96	**0.002**	41.25 ± 5.37	51.69 ± 6.55	**< 0.001**
O_2_/HR (unload)	ml/kg/beat	5.23 ± 0.69	6.19 ± 0.99	**0.001**	3.52 ± 0.55	4.83 ± 0.96	**< 0.001**
O_2_/HR (peak)	ml/kg/beat	10.92 ± 2.04	12.33 ± 1.83	**0.001**	6.77 ± 1.16	7.89 ± 0.88	**< 0.001**
△VO_2_/△WR	ml/(min*watt)	7.97 ± 0.93	8.91 ± 0.68	**0.004**	7.74 ± 1.37	8.70 ± 0.80	**0.022**
MET (unload)	–	2.17 ± 0.39	2.65 ± 0.49	**< 0.001**	1.93 ± 0.22	2.59 ± 0.44	**< 0.001**
MET (peak)	–	8.23 ± 1.00	9.52 ± 1.26	**0.001**	6.47 ± 1.13	7.61 ± 1.14	**< 0.001**
HR (unload)	bpm	94.07 ± 11.40	97.27 ± 11.18	0.324	97.50 ± 7.87	96.44 ± 9.33	0.620
HR (peak)	bpm	172.20 ± 9.17	176.13 ± 9.38	0.248	168.44 ± 14.60	169.94 ± 12.70	0.392
HRR	bpm	21.60 ± 9.52	20.67 ± 9.05	0.769	27.81 ± 13.44	26.69 ± 13.34	0.477
SpO_2_ (unload)	%	99.14 ± 0.95	99.00 ± 1.30	0.635	99.33 ± 1.18	98.80 ± 1.15	0.178
SpO_2_ (peak)	%	96.18 ± 2.56	98.36 ± 1.36	**0.043**	95.00 ± 2.26	96.42 ± 2.50	0.236
**Ventilatory function**							
VT (unload)	l	0.75 ± 0.17	0.8 ± 0.15	0.221	0.49 ± 0.09	0.62 ± 0.11	**0.001**
VT (peak)	l	1.94 ± 0.29	2.16 ± 0.37	**0.001**	1.22 ± 0.22	1.36 ± 0.21	**0.003**
RER (unload)	%	0.81 ± 0.09	0.82 ± 0.04	0.736	0.80 ± 0.07	0.78 ± 0.10	0.350
RER (peak)	%	1.22 ± 0.10	1.25 ± 0.09	0.408	1.18 ± 0.08	1.22 ± 0.08	0.161
BR% (unload)	%	90.20 ± 1.47	87.73 ± 1.94	**< 0.001**	89.88 ± 2.31	86.00 ± 2.78	**< 0.001**
BR% (peak)	%	52.07 ± 11.76	45.93 ± 10.44	**0.032**	60.19 ± 9.83	50.44 ± 8.16	**< 0.001**
**Gas exchange**							
V_E_/VCO_2_ (slope)	ratio	27.32 ± 3.66	24.92 ± 2.17	**0.031**	27.38 ± 5.35	27.24 ± 3.12	0.883
V_E_ (unload)	l	14.17 ± 1.91	17.95 ± 2.00	**< 0.001**	10.42 ± 2.36	14.63 ± 2.58	**< 0.001**
V_E_ (peak)	l	71.59 ± 16.83	82.02 ± 17.01	**0.015**	42.46 ± 10.09	53.95 ± 10.33	**< 0.001**
**CPET performance**							
Load_max_	watt	190.33 ± 33.54	193.8 ± 37.06	0.488	106.06 ± 19.25	109.26 ± 18.23	0.084
Exercise duration	sec	499.20 ± 76.16	502.40 ± 85.48	0.745	505.13 ± 79.74	515.69 ± 78.45	0.138
Borg’s scale	scores	13.07 ± 1.94	12.53 ± 1.64	0.424	13.06 ± 1.18	12.37 ± 0.96	0.081
Exercise duration	min	499.20 ± 76.16	502.40 ± 84.48	0.745	505.16 ± 79.74	515.69 ± 78.45	0.138

*P*_*1*_ stands for within-male group comparison between mask-on and mask-off conditions. *P*_*2*_ stands for within-female group comparison between mask-on and mask-off conditions. Significant results are indicated in bold

*Abbreviation*: *VO*_*2*_*/kg (peak)* Peak oxygen uptake per kilogram, *AT* Anaerobic threshold per kilogram, *O*_*2*_*/HR* Ooxygen pulse, *MET* Metabolic equivalent, *HR* Heart rate, *HRR* Heart rate reserve, *△VO*_*2*_*/△WR*, Oxygen uptake related to work rate, *BR%* Breathing reserve in percentage, *V*_*E*_ Ventilation, *VT* Tidal volume, *bpm* beat per minute, *CPET* Cardiopulmonary exercise test, *RPE scales* Rating of Perceived Exertion scale, *l* liter, *RER* Respiratory exchange ratio, *sec* second, *min* minute

### Secondary outcomes

Table 3 showed the gender differences on the CPET parameter changes (“△” was used to indicate the change between mask-on and mask-off condition). No significant differences in exercise tolerances and cardiac function as well as gas exchange were found between the male group and the female group. However, the male group showed significantly greater △VT (peak) and △BR% (peak) than the female group (△VT_peak_: male − 0.80 ± 0.15 l, female − 0.62 ± 0.11 l, *P* = 0.001; △BR%_peak_: male − 1.94 ± 0.29 l, female 1.22 ± 0.22 l, *P* < 0.001).

**Table 3:** Gender differences on the CPET parameter changes of wearing surgical masks

Parameters	Unit	Male group	Female group	***P*** value
**Exercise tolerance and cardiac function**				
△VO_2_/kg (warm up)	ml/min/kg	1.66 ± 1.32	2.38 ± 1.27	0.133
△VO_2_/kg (peak)	ml/min/kg	4.56 ± 4.45	4.07 ± 2.78	0.713
△VO_2_/kg (AT)	ml/min/kg	2.31 ± 2.20	3.94 ± 2.62	0.072
△VO_2AT_/VO_2max_	%	−2.40 ± 3.87	−0.13 ± 3.51	0.098
△O_2_/HR (warm up)	ml/kg/beat	0.96 ± 0.84	1.31 ± 0.85	0.266
△O_2_/HR (peak)	ml/kg/beat	1.41 ± 1.21	1.12 ± 0.82	0.442
△VO_2_/△WR	ml/(min*watt)	0.94 ± 1.05	0.96 ± 1.51	0.962
△MET (warm up)	–	0.48 ± 0.38	0.67 ± 0.35	0.161
△MET (peak)	–	1.29 ± 1.22	1.14 ± 0.89	0.686
**Ventilatory function**				
△VT (peak)	liter	−0.80 ± 0.15	−0.62 ± 0.11	**0.001**
△BR% (warm up)	%	0.05 ± 0.16	0.13 ± 0.12	0.118
△BR% (peak)	%	1.94 ± 0.29	1.22 ± 0.22	**< 0.001**
**Gas exchange**				
△V_E_ (warm up)	liter	3.77 ± 2.19	4.21 ± 2.30	0.596
△V_E_ (peak)	liter	10.43 ± 14.58	11.49 ± 7.71	0.801

*P* value stands for among group comparisons between male group and female group. Significant results are indicated in bold

*Abbreviation*: *VO*_*2*_*/kg (peak)* Peak oxygen uptake per kilogram, *AT* Anaerobic threshold per kilogram, *O*_*2*_*/HR* Oxygen pulse, *MET* Metabolic equivalent, *△VO*_*2*_*/△WR* Oxygen uptake related to work rate, *BR%* Breathing reserve in percentage, *V*_*E*_ ventilation, *VT* tidal volume, *CPET* Cardiopulmonary exercise test, *min* minute

Table 4 showed the within-group comparison of lower limb muscle activity (mean RMS) between mask-on and mask-off conditions in unload stage and incremental exercise stage, respectively. During the unload CPET phase, the results of sEMG showed that there were no significant differences of mean RMS between mask-on condition and mask-off condition in VM. RF, VL, TA, ST, BFCL, MG and LG. However, during the load CPET phase, the male group showed significant difference of mean RMS in VM between mask-on condition and mask-off condition.

**Table 4:** The within-group comparison of lower limb muscle activity (mean RMS) between mask-on and mask-off conditions in unload stage and incremental exercise stage, respectively

Low limb muscles	Unit	Male group			Female group		
		Mask-on	Mask-off	***P***_***1***_	Mask-on	Mask-off	***P***_**2**_
**Unload**							
VM	μV	16.29 ± 6.56	19.23 ± 9.73	0.223	20.31 ± 6.41	21.39 ± 8.77	0.638
RF	μV	24.64 ± 16.90	24.78 ± 15.39	0.952	25.41 ± 10.86	26.85 ± 7.84	0.611
VL	μV	28.87 ± 13.49	27.93 ± 11.61	0.791	31.59 ± 13.98	32.68 ± 12.31	0.593
TA	μV	50.54 ± 23.64	39.44 ± 10.58	0.055	52.09 ± 19.05	53.96 ± 16.73	0.728
ST	μV	18.92 ± 13.79	14.94 ± 7.91	0.207	22.56 ± 19.71	19.82 ± 9.20	0.587
BFCL	μV	21.20 ± 8.69	24.11 ± 7.67	0.265	25.75 ± 10.83	25.17 ± 11.01	0.876
MG	μV	24.76 ± 7.00	24.35 ± 8.76	0.856	29.91 ± 13.67	32.83 ± 17.19	0.462
LG	μV	32.00 ± 20.26	30.29 ± 15.49	0.755	33.14 ± 13.26	26.35 ± 10.60	0.140
**Load**							
VM	μV	65.36 ± 15.15	76.46 ± 19.04	**0.031**	51.50 ± 15.33	49.77 ± 17.10	0.556
RF	μV	53.72 ± 26.48	54.92 ± 27.67	0.800	39.01 ± 10.36	42.69 ± 10.76	0.279
VL	μV	75.19 ± 30.63	72.05 ± 15.36	0.721	68.21 ± 23.12	67.13 ± 17.74	0.792
TA	μV	60.26 ± 16.86	53.21 ± 13.70	0.074	57.04 ± 18.86	58.75 ± 18.61	0.778
ST	μV	35.89 ± 25.91	32.28 ± 14.02	0.474	36.58 ± 23.71	30.26 ± 9.17	0.242
BFCL	μV	35.68 ± 15.21	38.38 ± 10.45	0.384	35.85 ± 13.06	35.82 ± 11.27	0.994
MG	μV	30.86 ± 12.03	33.67 ± 16.13	0.380	34.84 ± 15.12	36.40 ± 21.54	0.698
LG	μV	36.46 ± 17.47	37.30 ± 15.84	0.854	35.30 ± 10.80	30.23 ± 12.01	0.257

*P1* stands for within-male group comparison between mask-on and mask-off conditions. *P2* stands for within-female group comparison between mask-on and mask-off conditions. Significant results are indicated in bold

*Abbreviations*: *RMS* Root means square, *VM* Vastus medialis, *RF* Rectus femoris, *VL* Vastus lateralis, *TA* Tibialis anterior, *ST* Semitendinosus, *BFCL* Biceps femoris caput longus, *MG* Medial gastrocnemius, *LG* Lateral gastrocnemius, *μV* microvolt

## Discussion

### Principal findings

This was a randomized cross-over study exploring the impacts of surgical masks on cardiopulmonary exercise capacity (by CPET) and lower limb muscle performance (by simultaneously sEMG), which was to further investigate based on our previous study [12]. Moreover, to our knowledge, this study is the first study to simultaneously measure sEMG data during CPETs and allocated the subjects considering the gender effect.

The main findings of this study are: wearing surgical masks has some negative effects on cardiopulmonary function in both male and female health subjects, although, based on self-rated scales, there was no difference in subjective feelings when comparing the mask-off and mask-on condition. Wearing surgical masks might produce a greater decrease in ventilatory function and lower limb muscle activity during high-intensity phase of CPET in male subjects than in female subjects.

### Comparison with other studies

First, wearing a surgical mask might have some negative effects on exercise tolerance and cardiac function, as reflected in the decreased VO_2_/kg, O_2_/HR, and △VO_2_/△WR after wearing a mask in both the male and female groups, which are related to the increased breathing resistance in the medical mask, requiring more work of the respiratory muscles and leading to higher oxygen consumption. However, Sven Fikenzer et al. [5] detected a similar cardiac output with and without mask during the incremental exercise test. This might be related to the greater age distribution and smaller subject size in Sven Fikenzer’s study (age 38.1 ± 6.2 years, nine males recruited subjects) [5]. This study also found out that no significant differences in HR and HRR in both male and female groups, which gave clue that monitoring HR alone is not the most sensitive indicator of exercise-related cardiac performance [17]. However, Lässing J et al. [18] found out that there was no significant difference in the change of blood pressure in exercise with mask on, but the peak heart rate of exercise was higher, the average cardiac output was higher, while Drivers’s study [19] indicated that the peak heart rate of wearing cloth mask for exercise test was lower, which might be related to the different test schemes. The former adopted constant power exercise test, and the latter adopted gradual treadmill exercise test, while this study adopted gradual incremental bicycle exercise test. Mehdi Ahmadian et al. [20] explored the effects of surgical mask, N95 mask and mask-off on hemodynamics and blood function in 144 healthy subjects. The results showed that there was no statistical difference in the changes of hemodynamics (systolic blood pressure, heart rate, rate pressure product) before and after exercise, regardless of gender, mask type and exercise intensity.

Second, the direct physical mechanism of wearing a medical mask is to affect ventilation and increased breathing resistance due to the 3-ply structure of non-woven material for preventing the spread of pathogenic organisms [21]. The related effect of wearing a surgical mask during different exercise conditions (unload and load) was the main aim of the study. The results showed increased breathing resistance and limitation of ventilation after wearing medical masks were found both in the unload and load phase that led to decreased VT and V_E_ as well as increased BR% and V_E_/VCO_2_ (slope), which was likely higher during the load phase. Lässing J et al. [22] (14 males, mean age 25.7 ± 3.5 years) confirmed a significant increase in respiratory tract resistance (0.58 ± 0.16 kPa l ^− 1^ vs. 0.32 ± 0.08 kPa l ^− 1^; *p* < 0.01) in patients wearing surgical masks. *p* < 0.01), and VE also decreased accordingly (77.1 ± 9.3 l min ^− 1^ vs. 82.4 ± 10.7 l min ^− 1^; *p* < 0.01). Shaw K et al. [23] considered that there was no significant effect on blood oxygen saturation, tissue oxygenation index and self-perceived fatigue in healthy young subjects wearing masks during intensive exercise, and that wearing masks was safe. Susan R et al. [24] reviewed the effects of wearing different masks on cardiopulmonary function through literatures, demonstrated that although the existing data indicated the negative impact of wearing cloth or surgical masks in healthy people could be ignored, and it would not significantly affect exercise endurance, but for people with severe cardiopulmonary conditions, any increase in resistance and / or small changes in blood gas may lead to dyspnea, thereby affecting exercise ability. So, further research is needed on the safety of wearing masks for long periods of time or during exercise in such subject groups. The study also found the bigger negative effect of ventilator function in wearing a mask during the load phase in the male group than in the female group, which might be related to more exercise intensity in male subjects.

Third, the study explored not only the direct cardiopulmonary effect of wearing a medical mask, but also the close related peripheral muscle performance by lower limb sEMG during CPET. We detected a decreased mean vastus medialis muscle activity after wearing medical mask compared to no mask in the male group. The sEMG results suggest that wearing a mask might decrease motor function, especially during high-intensity exercises, due to the dominant role of vastus medialis in cycling exercises [25]. Combined with the previous changes in cardiopulmonary function, the decline of exercise tolerance in healthy people caused by surgical masks, which may be caused by the peripheral mechanism. Because masks cause ventilation restriction to the reduction of oxygen intake, resulting in the decrease of oxygen intake in peripheral muscles and exercise tolerance. Although in Sven Fikenzer’s study, there was no significant change in blood lactate value as one of the main indicators of muscle metabolism before and after wearing medical masks [5]. Moreover, female subjects in the study showed no significant differences in muscle activities between the mask-on and mask-off conditions, which might also be related to the relatively lower exercise intensity compared with male subjects.

Finally, the study also showed no significant differences in subjective fatigue feeling based on Borg’s scales assessment after the CPET test in both the male and the female groups. However, the cardiopulmonary parameter changed between the mask-on and off conditions found in the study still suggested some negative effect on wearing medical masks during exercises.

### Limitations

There were three main limitations to this study. First, the blood test was not included in this study due to a previous study reporting no significant difference in peak blood lactate response when comparing the mask-on and mask-off conditions [5]. Furthermore, not immediately collecting arterial blood samples during or after CPET was also decided based on safety. The arterial blood pressure might be increased after strenuous exercise, which may cause difficulty in hemostasis. Second, our study only focused on the effect of wearing surgical masks on cardiopulmonary function because these masks are widely used and readily available in the community. Moreover, it is rarely possible to wear N95 masks for prolonged aerobic exercise due to the discomfort and community inapplicability. Third, elders and patients with cardiopulmonary diseases were not recruited in the study due to relatively little experience in this field. Further study should recruit various participants to extend the safety of application of wearing the surgical mask in daily life.

## Conclusions

Wearing medical/surgical mask showed a negative impact on the ventilation function in young healthy subjects during CPET, especially in high-intensity phase. Moreover, some negative effects were found both in ventilation and lower limb muscle actives in male young subjects during mask-on condition. Future studies should focus on the subjects with cardiopulmonary diseases to explore the effect of wearing mask.

## Abbreviations

AT: Anaerobic threshold per kilogram
BP: Blood pressure
BFCL: Biceps femoris caput longus
CPET: Cardiopulmonary exercise test
ECG: Electrocardiography
FEV1: Forced expiatory volume in 1 second
FVC: Forced vital capacity
HR: Heart rate
HRR: Heart rate reserve
LG: Lateral gastrocnemius
kg: Kilogram
MET: Metabolic equivalent
ms: Millisecond
MG: Medial gastrocnemius
PAR-Q: Physical Activity Readiness Questionnaire
RF: Rectus femoris
RMS: Root mean square
SpO_2_: Pulse oxyhemoglobin saturation
ST: Semitendinosus
sEMG: Surface electromyography
TA: Tibialis anterior
VO_2_/kg: Oxygen uptake per kilogram
VCO_2_: Volume of carbon dioxide released
V_E_: Ventilation
VL: Vastus lateralis
VM: Vastus medialis
VO_2 max_: Maximal oxygen uptake
VO_2_: Peak oxygen consumption
VT: Tidal volume
